# Construction of high quality Gateway™ entry libraries and their application to yeast two-hybrid for the monocot model plant *Brachypodium distachyon*

**DOI:** 10.1186/1472-6750-11-53

**Published:** 2011-05-19

**Authors:** Shuanghe Cao, Chamindika L Siriwardana, Roderick W Kumimoto, Ben F Holt

**Affiliations:** 1 Department of Botany and Microbiology, University of Oklahoma, 770 Van Vleet Oval, GLCH, Room 43, Norman, OK 73019, USA

## Abstract

**Background:**

Monocots, especially the temperate grasses, represent some of the most agriculturally important crops for both current food needs and future biofuel development. Because most of the agriculturally important grass species are difficult to study (e.g., they often have large, repetitive genomes and can be difficult to grow in laboratory settings), developing genetically tractable model systems is essential. *Brachypodium distachyon *(hereafter Brachypodium) is an emerging model system for the temperate grasses. To fully realize the potential of this model system, publicly accessible discovery tools are essential. High quality cDNA libraries that can be readily adapted for multiple downstream purposes are a needed resource. Additionally, yeast two-hybrid (Y2H) libraries are an important discovery tool for protein-protein interactions and are not currently available for Brachypodium.

**Results:**

We describe the creation of two high quality, publicly available Gateway™ cDNA entry libraries and their derived Y2H libraries for Brachypodium. The first entry library represents cloned cDNA populations from both short day (SD, 8/16-h light/dark) and long day (LD, 20/4-h light/dark) grown plants, while the second library was generated from hormone treated tissues. Both libraries have extensive genome coverage (~5 × 10^7 ^primary clones each) and average clone lengths of ~1.5 Kb. These entry libraries were then used to create two recombination-derived Y2H libraries. Initial proof-of-concept screens demonstrated that a protein with known interaction partners could readily re-isolate those partners, as well as novel interactors.

**Conclusions:**

Accessible community resources are a hallmark of successful biological model systems. Brachypodium has the potential to be a broadly useful model system for the grasses, but still requires many of these resources. The Gateway™ compatible entry libraries created here will facilitate studies for multiple user-defined purposes and the derived Y2H libraries can be immediately applied to large scale screening and discovery of novel protein-protein interactions. All libraries are freely available for distribution to the research community.

## Background

Use of the dicot annual *Arabidopsis thaliana *(Arabidopsis) as a model system continues to revolutionize our understanding of plant biology, but the development of alternative plant models promises to address numerous knowledge gaps. Especially relevant is the development of additional models for the agriculturally important grasses. Leading this charge is the emerging grass model system *Brachypodium distachyon *(Brachypodium). Brachypodium is a monocotyledonous, C3 temperate grass genus in the family Poaceae, subfamily Pooideae, which is closely related to wheat, oats, and barley [1,2]. Like Arabidopsis, Brachypodium has many advantageous features for genetic research, including small size, simple growth requirements, and a relatively small genome with diploid accessions [3,4]. While it would be preferable to directly study food crops (e.g., wheat) and biofuel crops (e.g., switchgrass), they are generally lacking in many of the desirable features that make Brachypodium such an excellent model system. Thus Brachypodium offers a relatively non-demanding entry point to study the agriculturally important grasses.

From a small handful of highly engaged individuals to the formation of the International Brachypodium Initiative (IBI), interest in using Brachypodium has steadily grown since it was first proposed in 2001 [5]. A major achievement for the IBI was the recently completed sequence of the *Brachypodium distachyon *(diploid accession Bd21) genome [6]. As happened with Arabidopsis 10 years earlier [7], completion of the Brachypodium sequence placed this model system on the fast track for many exciting discoveries. To facilitate these discoveries, many labs are actively building publicly shared tools. For example, the Brachypodium genome is well-annotated [6] and can be readily queried at several online sites, such as http://www.brachypodium.org. Numerous wild accessions have been collected and shared with the research community [8,9]. Supported by the underlying Bd21 genome sequence framework, these wild accessions represent abundant natural diversity for functional genetics studies. Essential methods for utilizing the genetic potential of Brachypodium are being rapidly developed and refined. For example, high efficiency Agrobacterium-mediated transformation [10-12] and optimized crossing techniques (http://brachypodium.pw.usda.gov/) were recently developed. Additionally, rapidly growing numbers of sequence indexed T-DNA insertion lines are now publicly available, allowing researchers to begin searching for loss of function alleles in specific gene targets ([13], http://brachypodium.pw.usda.gov/TDNA/). Therefore, many researchers are simultaneously converging on Brachypodium as a viable model system for studying the complex and agriculturally important grass lineage and most of the resource building efforts have resulted in publicly available tools that benefit the entire research community.

Complementary DNA (cDNA) libraries are a particularly useful resource that have been developed for Brachypodium [14]. cDNA libraries are essential tools for developing expressed sequence tag (EST) databases and exploring an organism's transcriptome. The development of highly efficient, *in vitro *recombination cloning technologies (e.g., Gateway™ technology) recently made it possible to capture cDNA libraries in entry vectors that allow easy shuttling to various downstream destination vectors [15-18]. In fact, the transfer and use of libraries originally cloned in recombination-ready entry vectors is only limited by the availability of suitable downstream destination vectors (or the willingness to create new vectors) [15,16,19,20]. A previous study comparing the shuttling of libraries by conventional ligation-based techniques versus recombination-based techniques demonstrated several major advantages of the latter approach: simplified directional cloning, reductions of chimeric clones, less size bias, and improved cloning efficiency [18]. Therefore, the use of recombination-based technologies to create cDNA libraries can greatly improve user convenience, potential downstream uses, and outcome quality.

One published example of a recombination-based methodology was the creation of high quality Arabidopsis cDNA libraries and the subsequent transfer of those libraries to yeast two-hybrid (Y2H) vectors [17]. Y2H screens require high quality libraries and are one of the most effective methods of identifying novel protein-protein interactions [21-26]. Most well established model systems have publicly or privately available Y2H libraries. Because the development of these libraries is time-consuming and technically difficult, labs may be precluded from attempting Y2H screens in the absence of suitable cDNA libraries in appropriate vectors. Generally, suitable libraries (both entry and destination) have large numbers of unique clones (>10^6^) and sufficient average clone lengths to ensure reasonable likelihoods of obtaining interacting partners with a variety of protein baits. Because of the previously mentioned qualitative features associated with recombinant cloning, this methodology is well-suited to the creation of Brachypodium cDNA libraries and their downstream shuttling to Y2H vectors.

Similar to the approach for Arabidopsis, we describe the creation of two Gateway™-ready Brachypodium cDNA libraries suitable for various downstream applications, including Y2H analyses. The two entry libraries represent Brachypodium mRNA populations collected from plants grown under different photoperiods (20-h and 8-h day lengths) or after eight different hormone treatments (hereafter referred to as the photoperiod and hormone libraries, respectively). Both libraries were cloned into the Gateway™ entry vector pDONR222 (Invitrogen) and were subsequently transferred to the Y2H "prey" vector pDEST22 (Invitrogen). Because our lab is interested in the functions of NUCLEAR FACTOR Y (NF-Y) transcription factors [27] and interactors are already described for a subset of these proteins [28-30], we isolated a Brachypodium NF-Y ortholog and used this as "bait" to perform initial proof-of-concept screens with both Y2H libraries. Demonstrating that the Y2H libraries perform very well, we were able to readily isolate known and novel interactors. Therefore, the Y2H libraries described here are immediately useful for protein-protein interaction studies and the cDNA entry libraries can be shuttled and adapted for many other downstream uses. All libraries, in their entry and Y2H vector forms, are available for free distribution to the research community.

## Results and Discussion

### Library Construction

Both libraries were constructed with the goal of maximizing the number of unique clones as opposed to constructing more narrowly representative "boutique" libraries. We reasoned that performing screens against multiple boutique libraries was not practical for most groups. Additionally, the actual creation and initial testing of each library is relatively complex and time consuming. Therefore, we opted to build two libraries - photoperiod and hormone - that each captured a relatively wide range of mRNA expression possibilities.

The photoperiod library represents mRNA from both long day (LD, 20-h light/4-h dark) and short day (SD, 8/16-h) grown shoots (entire aboveground plant) of two week old plants collected over a ~24 hour period. The LD cycle of 20/4-h was chosen because Brachypodium is rapidly induced to flower under these conditions [31] (as opposed to Arabidopsis where 16-h light is sufficient). The hormone library represents mRNA from both roots and shoots of plants treated with eight different hormones (ABA, ACC, BL, GA3, IBA, KT, MeJA, and SA - see Abbreviations), also collected over a 24-h post treatment period to ensure the capture of early and late response genes in the final library.

In addition to capturing Brachypodium mRNA from a range of times and treatments for each library, we also wished to maximize two important measures of library quality [17]: absolute numbers of independent clones and average clone length. To improve absolute numbers of primary clones, we used commercially available electrocompetent cells with transformation rates of >1 × 10^9 ^cfu/μg. To maximize clone length, we used a combination of high quality reverse transcriptase enzyme (SuperScript™ III, Invitrogen) followed by column chromatography (Sephacryl^® ^S-500 HR resin, Invitrogen) to size fractionate the cDNA population (i.e., avoid cloning truncated cDNAs and primers).

### Treatment confirmations

**Figure 1:**
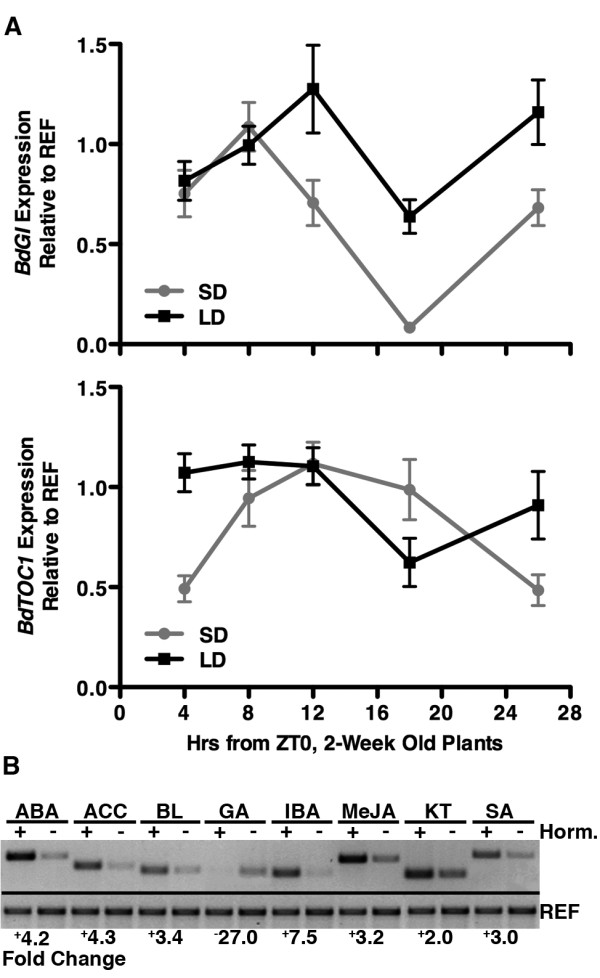
**Library treatment confirmations**. (A) Photoperiod library. SD and LD expression is shown for *BdGI *and *BdTOC1 *for the samples used to construct the photoperiod library (mean of four technical replicates/time point (±SEM)). Expression at each time point is relative to the REF gene (Bradi4g00660 [35], see Methods). Note that this should not be interpreted as a rigorous quantitative analysis of either gene, but is shown to demonstrate that samples used to construct the photoperiod library come from tissues experiencing varied light input with concomitant changes in gene expression. However, the SD and LD peaks of *BdGI *at ZT8 and 12, respectively, are similar to a previous report [32]. (B) Hormone library. Each treatment results in a clear change in the expression of the respective hormone marker gene. Quantitative values were generated by ImageJ (see Methods).

For each library, we confirmed that treatments correlated to expected transcriptional changes. For the photoperiod library, we examined the expression of Brachypodium orthologs for the genes *GIGANTEA *(*BdGI*, Bradi2g05230 [32]) and *TIMING OF CAB 1 *(*BdTOC1*/*PRR1*, Bradi3g48880 [33]; Figure 1A and Methods). These genes were chosen because they are either central to the circadian clock or act as markers for output from the clock - i.e., they are known to undergo differential peaks in expression throughout the circadian cycle [32,34]. As expected, both genes were expressed differentially during the sampled light conditions relative to an internal reference gene (Bradi4g00660, ubiquitin-conjugating enzyme [35], Figure 1A).

For the hormone library, we consulted the *Oryza sativa *(rice) literature (primarily microarray data) for appropriate hormone marker genes. After identifying promising rice marker genes for each hormone, we searched http://www.brachypodium.org for their respective Brachypodium orthologs. After testing two or three candidates for each hormone, suitable (hormone responsive) orthologous Brachypodium marker genes were found (Table 1 and Methods). Finally, we confirmed that all of these marker genes were activated (or repressed) by the appropriate treatment for the tissue samples used to construct the hormone library (Figure 1B). Collectively, this data shows that the mRNA isolated to construct the photoperiod and hormone libraries are representative of broad temporal and induced gene expression patterns.

**Table 1:** Marker genes for hormone treatment

Hormone Marker	IBI#	Os ortholog	At Ortholog	Common Name(s)	Function
**ABA**	Bradi2g22460	Os05g39690	At5G01670	None	NAD(P)-linked oxidoreductase superfamily
**ACC**	Bradi1g57590	Os10g11500	At4G33720	OsPR1#101	Pathogenesis-related 1 superfamily
**BL**	Bradi2g31700	Os05g15630	At4G03540	OsBLE3	Unknown molecular function
**GA3**	Bradi2g31760	Os05g19600	At1G20190	OsEXP3, AtEXPA11	Alpha-expansin gene family
**IBA**	Bradi2g50840	Os01g55940	At4G37390	OsGH3-2, AtAUR3	IAA-amido synthase
**MeJA**	Bradi3g30860	Os10g37340	At1G64660	OsRRJ1, AtMGL	Methionine gamma-lyase
**KT**	Bradi1g64920	Os03g18850	None found	OsPR10	Unknown, similarity to PYL, RCAR proteins
**SA**	Bradi2g05870	Os01g09800	At1G64280	NPR1, NIM1, SAI1	Local and systemic pathogen defense
**REF**	Bradi4g00660	Os12g44000	At5G42990	UBC18	Ubiquitin-conjugating enzyme

Where available, rice (Os) and Arabidopsis (At) orthologs, common names, and predicted functions are provided. See Methods for primers, additional information regarding each marker gene, and literature references used to find each marker.

### Library Quality

The Heyl Lab (Berlin, Germany) previously created four Arabidopsis Gateway™-ready libraries with, on average, ~7 × 10^5 ^primary clones [17]. Additionally, they reported the average clone length for one of their cDNA entry libraries (hormone) as 947bp. Because these Arabidopsis libraries have previously been screened with excellent results (after shuttling to a Y2H vector) [28,36], we set these quantitative measures as the desired benchmarks for quality of the Brachypodium libraries. The complete quality values for our hormone and photoperiod Brachypodium libraries are summarized in Table 2. Both Brachypodium libraries had ~5.5 × 10^7 ^total primary clones and average clone lengths of ~1,470 (hormone) and ~1,490bp (photoperiod). The major increase in average clone length for our Brachypodium libraries is likely partly due to significant improvements in reverse transcriptase enzymes over the past few years. In fact, we created an early test library using SuperScript™ II (instead of SuperScript™ III) and our average clone lengths were considerably shorter than those of the previously reported Arabidopsis libraries (data not shown). Further, we utilized column-based total RNA purification procedures (Methods); we find that columns generally give lower quantities, but higher quality RNA than TRIzol^®^-based methods. Based on our main two criteria, we conclude that these Brachypodium primary libraries are high quality.

**Table 2:** Measures of library quality

	Pre Recombinant Transfer		Post Recombinant Transfer	
	Primary Library (pDONR222)		Y2H Library (pDEST22)	
	Hormone	Photoperiod	Hormone	Photoperiod
**Total Prim. Clones**	5.47 × 10^7 ^	5.50 × 10^7 ^	1.0 × 10^7 ^	2.7 × 10^7 ^
	**Hormone (Kb)**	**Photoperiod(Kb)**	**Hormone (Kb)**	**Photoperiod(Kb)**
	
**Minimum Length**	0.50	0.30	0.45	0.20
**Maximum Length**	5.00	3.50	3.70	8.00
**Average Length**	1.47	1.49	1.42	1.58

Total primary clones were calculated from standard serial dilutions. For each library and vector, ~50 colonies were randomly chosen for plasmid isolation. Plasmids were digested and run on agarose gels to calculate respective insert sizes. Clone size differences between libraries are not considered significant (unpaired, two tailed t tests between pDONR222 and pDEST22 libraries: hormone library - P value = 0.7427, t = 0.3293 with 94 degrees of freedom; photoperiod library P value = 0.6213, t = 0.4955 with 104 degrees of freedom).

### Shuttling Libraries by In Vitro Recombination

We next examined the results of transferring primary libraries to a downstream destination vector. This was a simple, single-tube, *in vitro *recombination reaction using LR Clonase™ II to drive the transfer of primary clones from the entry vector (pDONR222) to the Y2H library destination vector (pDEST22). One major concern was the potential for size bias in these reactions - i.e., it was possible that larger, full length clones would be transferred with lower efficiency than less desirable, partial clones. It was previously demonstrated that the ability to capture larger clones in recombination-based cDNA libraries can actually be improved relative to conventional ligation-based reactions [18]. Additionally, for one of the previously described Arabidopsis libraries, no significant size bias was measured between the entry and destination libraries following the *in vitro *transfer process [17]. Because our average clone size was approximately 55% larger, we chose to reconfirm this finding for our Brachypodium libraries. From Table 2, average clone lengths are not significantly different after recombinant transfer. We did not attempt to test the outer limits of transfer efficiency for very large clones, but we note that the largest transferred clone (from a random sample of 50) was 8Kb in length. Thus, the efficiency of the reverse transcriptase reaction at amplifying longer templates might actually be the major concern where larger clones are highly desirable in a library.

Our results both confirm previous studies and extend them to libraries with larger average clone lengths [17,18]. It remains unclear whether or not specific entry vector/destination vector recombination reactions might affect the efficiency of insert transfer, but this will need to be tested by the end user on a case-by-case basis. For the purposes of creating the Y2H libraries described here, we demonstrate that there are no significant differences in the average clone lengths after recombination between pDONR222 cDNA entry libraries and pDEST22 destination vectors.

### Proof-of-concept library screens

Although the quality features of both Y2H libraries appear excellent, the most important measure is the ability to effectively isolate interacting proteins. The best protein choice for initial proof-of-concept screens is one with well-described interacting partners. In this way one can quickly determine whether or not screening each library yields expected interactors. For choosing such a candidate, there is currently very little published data regarding particular Brachypodium proteins and their known interactors. Nevertheless, we could infer likely protein-protein interactions from Arabidopsis.

Because our lab is particularly interested in the functions of NF-Y proteins and we have developed Arabidopsis Y2H interaction datasets for several of these proteins, we decided to use their Brachypodium orthologs for initial proof-of-concept screening. We have previously demonstrated that Arabidopsis NF-Y, subunit C3, 4, and 9 (NF-YC3, 4, and 9) proteins readily interact with various NF-YB and CCT (CONSTANS, CONSTANS-LIKE, TOC1) proteins in Y2H screens and by *in vivo *immunoprecipitation [28]. Additionally, using phylogenetic, genetic, and biochemical approaches, we and others have extended these results to demonstrate that NF-YB, NF-YC, and CCT proteins cooperatively function in protein complexes to control photoperiod-dependent flowering [27-30]. Therefore, using NF-YC as the bait protein fulfilled our criteria for good proof-of-concept screening material.

**Figure 2:**
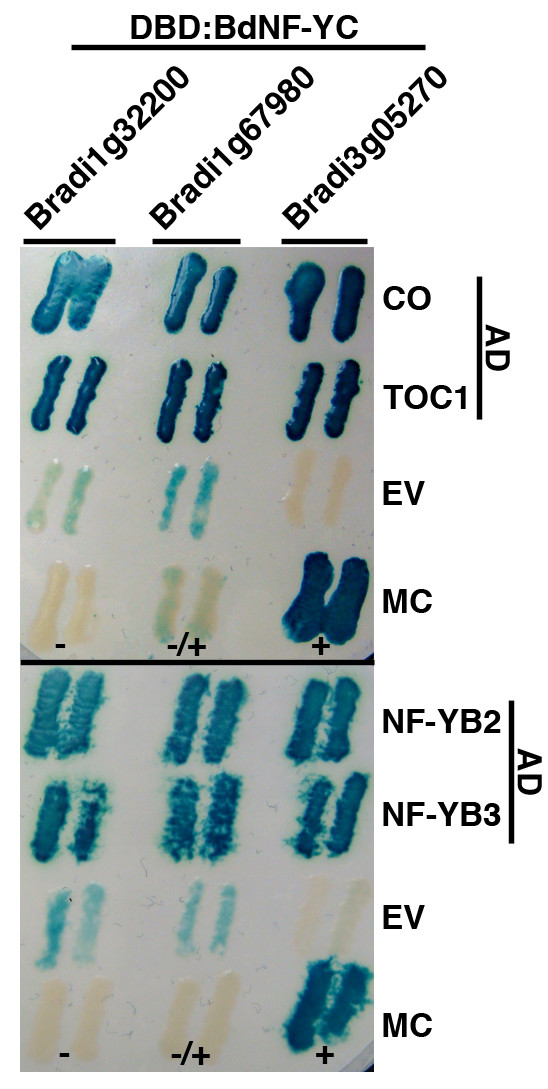
**Y2H direct interaction assays**. The Brachypodium NF-YC orthologs Bradi1g32200, Bradi1g67980, and Bradi3g05270 were cloned as Gal4 DNA binding domain (DBD) fusions and directly tested for the ability to physically interact with previously described Gal4 activation domain (AD) fusions to full length Arabidopsis CO, TOC1, NF-YB2, and NF-YB3 [28]. EV - empty vector; MC - manufacturer's (Invitrogen) controls (+ strong interactor, +/- weak interactor, - non-interactor).

To perform this screen, we first used BLAST and phylogenetic analyses to identify three Brachypodium orthologs of Arabidopsis NF-YC3/4/9 (Bradi1g32200, Bradi1g67980, and Bradi3g05270). To demonstrate that these proteins would, at least theoretically, be good choices to screen the Brachypodium Y2H libraries, we initially performed directed Y2H tests against already cloned Arabidopsis NF-YB (NF-YB2 and NF-YB3) and CCT (CONSTANS (CO) and TOC1) proteins (Figure 2). From Figure 2, the Brachypodium NF-YC orthologs all effectively interacted with a panel of previously cloned Arabidopsis NF-YB and CCT proteins. However, Bradi1g32200 and Bradi1g67980 both displayed low levels of autoactivation (see the "EV" or empty vector interaction rows). Because Bradi3g05270 did not display any obvious autoactivation, we chose this protein to perform the actual proof-of-concept screens against the Brachypodium libraries.

Using Bradi3g05270 as bait, 62 and 73 interacting clones were isolated from the Brachypodium hormone and photoperiod libraries, respectively. As expected, we readily isolated BdNF-YB and CCT protein interactors from both libraries (Table 3). However, the distribution of interacting proteins was substantially different between libraries. Only one CCT protein (2%) was isolated in the hormone library screen, while this same class represented 51% of the interactors from the photoperiod library. Likewise, the BdNF-YB protein Bradi3g15670 represented 31% of the hormone library interactors, but was not isolated from the photoperiod library. While it is tempting to speculate about biological meanings for these types of results (e.g., is Bradi3g15670 activated strongly by one of the hormone treatments?), caution is advisable as Y2H results can be highly variable between screens. This can be true for the same bait sequentially screened against the same library [37]. The results here are similar to a screen we performed using NF-YC9 as bait against an Arabidopsis library [28], although we found a higher percentage of novel interactors with the Brachypodium libraries (35% and 19% for the hormone and photoperiod libraries, respectively, versus 6% for the previous Arabidopsis screen). Overall, these results clearly demonstrate that expected and novel interactors can be readily isolated from these two libraries.

**Table 3:** Proof-of-concept results for the Brachypodium hormone and photoperiod libraries

	Photoperiod Library		Hormone Library	
	
Interactors	# Isolated	Percent	# Isolated	Percent
**NF-YB Family**				
Bradi1g21900	17	23	12	19
Bradi2g54200	5	7	8	13
Bradi3g15670	0	0	19	31
**CCT Family**				
Bradi1g11310	11	15	0	0
Bradi2g14220	6	8	0	0
Bradi3g05800	1	1	1	2
Bradi3g33340	3	4	0	0
Bradi3g41500	*11	15	0	0
Bradi5g14600	5	7	0	0
**Novel Interactors**	14	19	22	35

**Overall Totals**	73	100	62	100

*6/11 blast hits for Bradi3g415000 were short (25-60bp) matches, but the other five were long matches (~380 bp perfect match).

Annotations as NF-YB or CCT proteins came from direct nucleotide blasts (blastn) of Y2H Bradi3g05270 interactors at the Brachypodium database (http://www.brachypodium.org) and/or blastx at The Arabidopsis Information Resource (TAIR, http://www.arabidopsis.org).

## Conclusions

We have developed two Brachypodium Gateway™ cDNA entry libraries corresponding to both photoperiod and hormone treatment time courses. Both libraries have the hallmarks of any high quality cDNA library: many primary clones (~5.5 × 10^7^) and excellent average clone length (~1.5 Kb). Because these libraries were generated in a Gateway™ vector, they have many potential downstream uses. The entry libraries were additionally transferred by recombination-based techniques to create two unique Y2H libraries. Proof-of-concept screens using the two Y2H libraries demonstrate that expected interactors can be readily isolated against a known bait. We note that a subset of the novel interactors isolated in the Brachypodium screen are likely to represent parallel interactions in Arabidopsis and other plant species. Therefore, researchers who do not work with Brachypodium might also find generalizable value in screening these libraries with their favorite Brachypodium ortholog.

All four libraries - i.e., the entry cDNA libraries pDONR222-Hormone and pDONR222-Photoperiod and the Y2H libraries pDEST22-Hormone and pDEST22-Photoperiod - have been carefully amplified using a reduced bias, semi-solid agarose method [38] and are freely available for distribution to the research community (user pays shipping). Libraries will be mailed as lyophilized pellets with brief instructions on reconstitution, and the recommended screening procedure for Y2H libraries. There is no MTA associated with the distribution of these libraries, although end-users are requested to cite this publication if they use these libraries for published research. Material requests should be addressed to the corresponding author, Ben Holt (benholt@ou.edu), with the email subject "Brachypodium Library Request" and a short message stating which libraries you wish to receive. We encourage the recombination-based transfer of these Gateway™ cDNA entry libraries to unique vectors and request that any resulting libraries be freely shared with the community.

## Methods

### Plant sample preparation and mRNA extraction

The community standard diploid inbred line of *Brachypodium distachyon*, Bd21, was used for generation of both cDNA libraries. Seeds were initially de-husked and sterilized by soaking in a solution of 25% household bleach (5.25% NaOCl) supplemented with 0.1% Tween20 for 30 min with occasional rocking. Sterilized seeds were thoroughly rinsed four times with sterile water and then placed on sterile Murashige and Skoog (MS) agar (4.4 g/l MS salts with vitamins, 3% sucrose, pH 5.8, and 0.4% phytagel). To provide sufficient vertical space for two weeks of plant growth, we used Greiner Bio-One disposable plant culture containers (distributed by VWR). Seeds were cold-treated at 4°C for four days and then transferred to a Conviron ATC13 growth chamber growth chamber at 22°C, 150µE light intensity (standard florescent bulbs).

For the hormone library, plants were grown for two weeks in 16-h light/8-h dark (16/8) cycles. For each hormone, 12 plants were hormone treated by spraying the leaves to runoff and allowing the residual hormone solution to soak into the growth media. The hormones used and concentrations were as follows: ABA (100µM) ACC (50µM), BL (50µM), GA3 (50µM), IBA (50µM), KT (50µM), MeJA (100µM), and SA (50µM). Three plants (root and shoot) were collected for each hormone treatment at 4, 8, 14, and 24-h after treatment, totaling 96 individual plants. For the photoperiod library, plants were grown in 20/4 or 8/16-h light cycles for 14 days. Shoot tissues were collected at zeitgeber (ZT) 4, 8, 12, 18, and 26. For each time point and light cycle, 8-10 plants were collected, totaling ~75 plants. All tissues were flash frozen in liquid nitrogen and stored at -80°C until mRNA extraction was performed.

To extract RNA, each hormone/time point sample was ground in a frozen mortar and total RNA was individually extracted using the E.Z.N.A. Plant RNA Kit (Omega Biotek). Quality and quantity of extracted RNA from all samples was confirmed by both agarose gel visualization and spectrometry (NanoDrop™ 1000, Thermo Scientific). mRNA was further extracted from the total RNA fraction using the MultiMacs™ mRNA Isolation Kit following the manufacturer's instructions (Miltenyi Biotec). After completing purification of each mRNA sample (each condition and time point), equal quantities of mRNA from each sample were combined and used in the construction of each library (see below).

### Construction, qualification and amplification of the entry cDNA library

Following mRNA purification and first strand cDNA synthesis, we used the Cloneminer™ II cDNA Library Construction Kit per the manufacturer's instructions to create the entry libraries in pDONR222 (Invitrogen). We experimented with both suggested options for size fractionation of clones - gel purification and Sephacryl^® ^S-500 HR resin column size chromatography. We found better results using the column chromatography and both libraries were constructed in this manner. Resulting libraries were transferred to DH10B™ T1 phage-resistant electrocompetent *E. coli *by electroporation (12 reactions/library, 25µl/reaction). Small aliquots were set aside to perform serial dilutions and calculate the number of primary clones. The remaining transformed cells were diluted with storage solution as per the Cloneminer™ II manual. To examine average clone lengths, we isolated plasmid DNA by standard methods for ~50 randomly selected clones from each pDONR222 library. We then digested the plasmids with the restriction enzyme BsrGI (Fermentas) and calculated the size of all fragments, subtracting the vector backbone (summarized in Table 2, gel images not shown). To examine average clone lengths for the pDEST22 (Y2H) libraries, we performed double digestions on the individual clones with MluI and SacI (Fermentas). Prior to recombinant shuttling and for bulking/distribution purposes, all libraries were amplified by a semisolid agarose amplification method that reduces bias in the resulting populations [38]. A simplified version of this method can be found in the pCMV-Script XR cDNA Library Construction Kit manual at http://www.genomics.agilent.com/files/Manual/200465.pdf. Transferring the entry library from pDONR222 to the Y2H destination library (pDEST22) was done in a single tube recombination reaction using LR Clonase™ II following the manufacturer's instructions (Invitrogen). The reaction was 3µl pDONR222 library (50ng/ µl), 3 µl pDEST22 vector (150ng/µl), 8µl 1x Tris/EDTA (TE) Buffer (pH 8), and 6µl LR Clonase™ II enzyme mix. The reaction was incubated at 25°C for 20-h and then transformed into 6 tubes of electrocompetent cells (50µl/reaction).

### Confirming treatment effects

We used reverse-transcription, polymerase chain reaction (RT-PCR) with known marker genes (rice orthologs, see below) to determine that hormone treatments worked. For the RT-PCR reactions, a sample of total RNA from each treatment (untreated and four hours post-treatment) was first digested at 37°C for 30 minutes with recombinant DNase I (Ambion/Applied Biosystems). cDNA was then generated using the RT enzyme Superscript II (Invitrogen) per the manufacturer's instructions. cDNA samples from each hormone treatment and the respective untreated controls were used in standard PCR reactions.

To identify the Brachypodium marker genes corresponding to specific hormone treatments, we primarily referred to hormone treatment microarray data from various rice publications. The complete reference list by hormone is as follows: ABA [39], ACC [40], BL [41], GA3 [42], IBA [43], MeJA [44], KT [45], SA [46], REF (UBC) [35]. We selected good candidates from the rice data and queried the Brachypodium database for suitable orthologs using BLAST tools at two web sites: http://www.plantgdb.org/BdGDB/cgi-bin/blastGDB.pl and http://blast.brachypodium.org. We then used Geneious Pro 5.1.4 to design primers for each marker (Table 4). We report here the best primer pair/marker gene we identified for each hormone treatment, although we typically tried two or three markers per hormone (data not shown). For a uniformly expressed control gene, we used the Brachypodium ubiquitin-conjugating enzyme Bradi4g00660 [35]. To quantify differences in expression, gel images were imported into ImageJ [47] and volume reports were created for each gene under non-induced and hormone-induced conditions. Expression of each marker gene was normalized against Bradi4g00660 expression from the same cDNA sample, followed by calculating post hormone expression (induced/non-induced). In the same manner, we used RT-PCR on the *BdGI *and *BdTOC1 *[32,33] to determine that our photoperiod library was cycling as expected.

**Table 4:** Information for amplifying hormone and photoperiod marker genes

Photoperiod	IBI#	Forward primer (5'-----3')	Reverse primer (5'-----3')	Product (bp)
***BdGI***	Bradi2g05230	**TCTTGCGTGCAATTGGAACT**	**AGCAACGTTGGCTGAGACAG**	100
***BdTOC1***	Bradi3g48880	**TAGTCGCTCTGACAGGAGGGCTG**	**TAACAAACTGCCCTCTCACCCTTG**	90

**Hormone**	**IBI#**	**Forward primer (5'-----3')**	**Reverse primer (5'-----3')**	**Product (bp)**

**ABA**	Bradi2g22460	**ACCCCAGGGCAGGTGCTCATTAAGT**	**CTCCTCTCCGGTCAGGACACGCTTC**	180
**ACC**	Bradi1g57590	**CATTACACGCAGGTGGTGTGGAGGA**	**TAGGGGCTCTCCCCGTTGAAGTTCC**	132
**BL**	Bradi2g31700	**CATCTGCGGGCAGGTGCAGGACTAC**	**CGATGACGTGGAGGGAGTACATGATG**	116
**GA3**	Bradi2g31760	**GTGGTGGCGCAGATGTGGATCAAGG**	**GAGAGGCTCTGCCCGTTGAGGTAGG**	110
**IBA**	Bradi2g50840	**ATTGGACCGCTGGAGATCAGGGTGGTG**	**GGCGCCTTGTACTGGTTGATGGACG**	101
**MeJA**	Bradi3g30860	**CACCACCTCCAGAACACCACCCAGT**	**GTAGCCCACGGACATGCGGATAAGC**	165
**KT**	Bradi1g64920	**GGCGTGGAGGTGAAGGACGAGATCA**	**ATGCTTCCGGATTGGCGACGAGGTA**	97
**SA**	Bradi2g05870	**CTCGGTAAACGCTTCTTTCCGCGCTG**	**ACCGGGCAAACTCCTCCTTGTCCTC**	185
**REF**	Bradi4g00660	**GGAGGCACCTCAGGTCATTT**	**ATAGCGGTCATTGTCTTGCG**	193

### Yeast two-hybrid - direct interaction tests and library screening

Full-length coding regions of *NF-YB2, NF-YB3, Bradi1g32200, Bradi1g67980*, and *Bradi3g05270 *were directionally cloned into the Gateway™ entry vector pENTR™/D-TOPO^® ^as described by the manufacturer (Invitrogen, cloning primers and pENTR clones available upon request). *CO *and *TOC1 *clones (stock# GC105432 and G21896, respectively) were obtained from the Arabidopsis Biological Resources Center in the pENTR223 vector. These entry vector clones were then transferred to appropriate Y2H vectors to perform direct interaction assays (or library screens for Bradi3g05270). All direct interaction assays and library screens were performed as described in the ProQuest™ manual (Invitrogen). Library screens were performed with 10mM 3AT (3-Amino-1,2,4-Triazole) to prevent false positive detection due to any low level autoactivation by the DBD:Bradi3g05270 fusion protein. For X-Gal assays (Figure 2), yeast colonies were transferred to nitrocellulose membranes and incubated in Z-buffer containing X-Gal (5-Bromo-4-chloro-3-indoxyl-beta-D-galactopyranoside, Gold Biotechnology) after freezing in liquid nitrogen, as described in the ProQuest™ manual.

## List of abbreviations

ABA: abscisic acid; ACC: 1-aminocyclopropane-1-carboxylic acid; BL: brassinolide; GA3: gibberellic acid A3; IBA: indole-3-butyric acid; KT: kinetin; MeJA: methyl jasmonic acid; SA: salicylic acid.

## Authors' contributions

BFH and RWK conceived and directed the project. SC was primarily responsible for the research and experimental procedures performed. CLS performed all of the directed Y2H assays and assisted SC in library proof-of-concept assays. BFH primarily wrote the manuscript with assistance from RWK, SC, and CLS. All authors have read and approved the final manuscript.
